# The impact of public health expenditure and gross domestic product per capita on the risk of catastrophic health expenditures for OECD countries

**DOI:** 10.3389/fpubh.2023.1122424

**Published:** 2023-04-06

**Authors:** Selma Söyük

**Affiliations:** Department of Health Management, Faculty of Health Sciences, Istanbul University-Cerrahpaşa, Istanbul, Türkiye

**Keywords:** catastrophic health expenditures, health spending per capita, income level, panel data, regression

## Abstract

**Introduction:**

Catastrophic health expenditure refers to situations where households face financial ruin due to high healthcare costs. For household spending on health services, the lack of pre-payment mechanisms to equalize the low payment capacity and risk, and the inability of countries' health financing systems to fulfill their duties adequately all contribute to the creation or increase of the risk of catastrophic health expenditure. This situation has devastating effects on poor households first, but if the prevention mechanisms are insouciant, it can threaten the health system of the entire country. The research aims to assess the impact of the pre-paid financing model implementations and income levels on the ability of countries to reduce the risk of catastrophic health expenditure.

**Methods:**

The paragraph explains the data used in the study, which is taken from OECD countries between 2003 and 2019. It also mentions the statistical models used in the study, which are static and dynamic panel regression models.

**Results:**

The findings indicate that pre-paid financing models, such as those based on taxation, can help reduce the risk of catastrophic health expenditure. The study also reveals that income levels play a role in this regard, with countries with higher incomes being better able to reduce the risk of catastrophic health expenditure.

**Discussion:**

The study suggests that healthcare financing systems should aim to provide effective services and financial protection to improve universal health coverage and reduce the risk of catastrophic health expenditure. Further researches using different health indicators and inputs could add to the existing literature on how to limit catastrophic health expenses and address other related questions.

## Introduction

Following the United Nations Millennium Declaration adopted in 2000 with the participation of 189 countries, the determined targets were transformed into an action plan. The goals in the development and poverty eradication section of the declaration are to reduce poverty and hunger, combat ill health, gender inequality, lack of education, lack of access to clean water, and environmental degradation. Goals in the field of health include reducing child mortality, improving maternal and child health, and combating HIV/AIDS, malaria, and other diseases. Improving health is central to the millennium development goals because poverty negatively impacts health and poor health leads to loss of income and catastrophic health expenditures (1). Along with improved health status and responsiveness, fair financing is one of the primary goals of the healthcare system. The World Health Organization (WHO) has identified three main goals for health systems: (1) Improving the health of populations -better health status- (2) Improving the responsiveness of the health system to the population it serves -responsiveness- (3) Fairness in financial contribution i.e., the extent to which the fair distribution the burden of paying for the health system is across households -fair financing- (2).

WHO has defined universal health coverage (UHC) as a mechanism that guarantees equitable access to basic promoting, preventive, curative, and rehabilitative health interventions for all citizens at an affordable cost, thereby ensuring access equality. Healthcare financing is a health system function that mainly serves universal health coverage by providing effective service and financial protection (3). The healthcare financing system aims to protect households from financial risk due to illnesses. This goal is also well-articulated in the world health organization 2010 report as the UHC goal (4, 5). UHC refers to a situation where all people can obtain needed health services at a good level of quality without suffering undue financial hardship (6). The effect of the lack of protection mechanisms is not just that people can suffer the burden of the illness but also the economic ruin and impoverishment of financing their care, yielding increased poverty in the short and long run (7). So, health systems must ensure that individuals have adequate financing mechanisms for acquiring preventive and curative care without deepening into catastrophic health expenditures (CHEs) and poverty (8).

Three factors must be present for catastrophic expenditures to arise; the presence of health services requiring out-of-pocket payments, low household capacity to pay, and lack of prepayment mechanisms for risk pooling. Out-of-pocket costs include all health-related expenditures that households make while receiving services, such as examination fees, purchase of medicines, materials, or devices, and hospital bills. The definition of household paying capacity is the non-subsistence expenditure of the household. Subsistence expenditures include basic needs such as food, shelter, and clothing. Prepayment refers to the situation where funds for health are collected through taxes and/or insurance contributions (9).

CHEs occur when out-of-pocket health payment as a share of the household income or capacity to pay exceeds a predetermined threshold level (10). Catastrophic expenditure is defined as “a morbid condition that results in health care costs that exceed a person's income, or which compromise financial independence, reducing him/her to subsistence or near-poverty levels” (11).

Catastrophic healthcare expenses are not solely due to expensive medical procedures or treatments. Just as a minor health expenditure can force a low-income household to cut back on essential expenses such as food, housing, or education, significant health expenses can lead to financial ruin and bankruptcy for wealthy individuals and families (12). Therefore, catastrophic healthcare expenditures are seen in low-income countries and high-income groups (10). While there is no consensus on the exact threshold for defining a catastrophic expenditure, most agree that it should be based on a household's ability to pay (11).

There are two different methods for calculating catastrophic health expenditures; the first is based on expenditure, and the second is on the income approach. According to the expenditure approach, catastrophic health expenditure occurs when out-of-pocket health expenditure exceeds a certain point of the total expenditure other than the basic expenses made by individuals to sustain their lives. The generally accepted rate is between 45 and 55%. However, because of deficiencies in calculations, this approach has been criticized (13). On the other hand, WHO has defined catastrophic health spending as the out-of-pocket health care expenditure of the household exceeding 40% of the household payment capacity (9). According to the income-based approach, catastrophic health expenditure occurs when out-of-pocket health expenditure exceeds some portion of the household income. In the literature, the most used threshold is 10% of yearly income when the denominator is total expenditure. That represents an approximate threshold at which the household is forced to sacrifice other basic needs, sell productive assets, incur debt, or become impoverished (14).

In addition to enabling people to access care when needed, national health financing systems must shield households from financial disasters by reducing out-of-pocket expenditures. But catastrophic expenses do not automatically disappear with increased income. In the longer term, the aim should be to develop prepayment mechanisms such as social health insurance, tax-based financing of health services, or some mix of prepayment mechanisms. In this direction, this research aims to examine the effects of countries' prepaid financing model implementation and income levels on their capacity to reduce the risk of catastrophic health expenditures.

## Materials and methods

This paper investigates the impact of prepayment financing models, in other words, the extent and existence of public health expenditures and income on the capacity to reduce the risk of catastrophic health expenditures with static and dynamic panel regression analysis for 34 OECD countries from 2003 to 2019.

Panel data are multidimensional data containing measurements over time. It covers observations of multiple phenomena in more than one-time period for the same organizations, individuals, or countries. In panel data consisting of *N* units, and *T* number of observations, *N* and *T* are higher than one (15). The simultaneous use of time and unit dimensions in panel data makes many data analyses usable by increasing the degrees of freedom. The panel data regression model is generally defined as follows:


$$\begin{array}{l} Y_{it}=\beta_{0it}+\beta_{1it}X_{1it}+\beta_{2it}X_{2it}+\;.\;.\;.+\beta_{kit}X_{kit}+u_{it}\\ \;\;\;\;\;\;\;\;\;\;\;\;\;i=1\;\;N,t=1\;\;T. \end{array}$$

In the study, the probability of countries making catastrophic expenditures when a surgical procedure is needed, representing the dependent variable, catastrophic expenditure, was taken as a proxy indicator. The proxy indicator of the income level is the income level in dollars according to the state domestic product (SDP) per capita, and the total out-of-pocket health expenditure per person in the relevant year, representing out-of-pocket expenditure. The study examined the health systems (public premium financing, tax financing, and private financing) of different countries to assess the impact of their prepayment mechanisms. Grouping takes into account the presence or absence of a prepaid model in the country's health services, which are primarily financed by public premiums and taxes. For this, the existence of a prepaid model is accepted as being 70% above the weighted average of public health expenditures among health expenditures.

The data consists of annual data between 2003 and 2019. For this reason, panel data analysis was used. Israel and New Zealand were excluded from the data set and analyses due to missing data from the 36 OECD countries, resulting in a total of 34 countries being studied. STATA 13.0 was used for all estimations.

### Dataset and model

The gross domestic product based on purchasing power parity (PPP) per capita GDP as an income-level proxy and domestic general government health expenditure (% of current health expenditure) were used as an indicator along with the risk of catastrophic health expenditures. The share of the risk of catastrophic health expenditures for surgical procedures, public health expenditures and income level data were accessed from the World Bank database. It was used a panel model controlling fixed and time effects and used also dynamic model (Generalized Moments Method-GMM) due to the endogeneity ().

The fixed effects regression model used in the research is as follows:


$$\begin{array}{l} che_{it\;}=\;\beta_{0}+\;\beta_{1}pre_{it}+\beta_{2}income_{it}+u_{it} \end{array}$$

The dynamic model regression model used in the research is as follows:


$$\begin{array}{l} che_{it\;}=\;\beta_{0}+\beta_{1}che_{it-1}+\;\beta_{2}pre_{it}+\beta_{3}income_{it}+u_{it}\; \end{array}$$

In the above equation: β is for the independent coefficients, *i* is for the countries, and *t* shows the time. The source of the data in the model, descriptive statistics, and other necessary explanations are given in Table 1.

**Table 1:** Explanations about variables in the model and descriptive statistics.

**Variable**	**Definition**	**Source**	**Mean**	**Min**.	**Max**
che	The proportion of population at risk of catastrophic expenditure when surgical care is required for i country at t time.	World Bank Data (2022). https://data.worldbank.org/indicator/SH.SGR.CRSK.ZS	10.61	0.0	96.8
pre	Domestic general government health expenditure (% of current health expenditure) for i country at t time	World Bank Data (2021). https://data.worldbank.org/indicator/SH.XPD.GHED.GE.ZS	70.5	29.7	87.63
income	Gross domestic product based on purchasing-power-parity (PPP) per capita GDP for i country at t time.	World Bank Data (2022). https://data.worldbank.org/indicator/NY.GDP.PCAP.PP.CD	37,416	9,587	11,7341

In the study, the countries whose data are available for the period between 2003 and 2019 were included and used a balanced panel data method. The reason why the data were cut in 2019 in the study is that the data for the last year announced for all the variables included in the model is 2019. In other words, 2019 is the most recent data.

## Results

The study sample consists of annual data from 34 OECD countries between 2003 and 2019. Before the model prediction for static regression modal results, the model's structure was tested to reach more accurate results in the study. The presence of time and/or unit effects in the model was tested with F and LR tests to see whether the model was classical regression. The least squares estimator, a classical regression estimator, gives biased results in the presence of unit or time effects. For this reason, in panel regression models, it should be tested first whether there is a unit or time effect in the model. The null hypothesis of the F and LR tests states that there is no unit or time effect, while the alternative states that there is a unit or time effect (15).

Researchers should investigate the relationship between the unit/time effects in the model and the independent variables. If there is a correlation between the independent variables and the unit/time effect in the model, researchers should use a fixed effects model. In this case, where the use of fixed effects model estimators is appropriate, if there is no relationship between the unit effects in the model and the independent variables, it would be more appropriate to use random effects model estimators instead of the fixed effects model. In the presence of unit/time effect, Hausman Test is used to test the relationship between these effects and independent variables (15).

Table 2 shows the results of the F, LR, and Hausman tests, performed before model estimation to determine the model type. The fact that the F and LR test results were statistically significant at 1% means that there is at least one time or unit effect in the model, and therefore it is understood that the classical model estimation is not suitable for this case. Both F and LR tests gave valid results for the one-way unit effect in the model seen in Table 2. In the effect of the unit effect, it should be decided whether the effect is fixed or random, that is, whether the E(α_i_, x_it_ = 0) condition is obtained by the Hausman test.

**Table 2:** F, LR, and Hausman test results.

**Model**	**F test**	**LR test**	**Hausman test**
*che*_*it*_ = β_0_+ β_1_*pre*_*it*_+β_2_*income*_*it*_+*u*_*it*_	F_unit_ = 1,654 (0.000)	LR_unit_ = 2,052.65 (0.000)	H_test_ = 16.90 (0.000)
	F_time_ = 0.001 (0.999)	LR_time_ = 0.002 (0.999)	
	F_unit − time_ = —	LR_unit − time_ = ——–	
Hypotheses	Unconstrained Model: Y_it_ = X_it_β + μ_i_ + u_it_ Restricted Model: Y_it_ = X_it_β + u_it_ H_0_ = μ_1 =_ μ_2 = ……. =_ μ_N − 1_ = 0	LR = −2[l(Limited)–l(Unconstrained)] H_0_ = ∂_*u*_ = 0	H_0_ = “there is no correlation between explanatory variables and error term” The random effects model is consistent H_1_ = “there is correlation between explanatory variables and error term” The fixed effects model is consistent
Result	H_0_ has been rejected. One-way unit domain	H_0_ has been rejected. One-way unit domain	H_0_ has been rejected. Fixed effects model is appropriate

Hausman test is used to make a valid choice between fixed effects and random effects, examining whether the difference between the parameter estimators of the fixed-effect model and the parameter estimators of the random model is statistically significant (16, 17). Since the null hypothesis of the Hausman test for the model was rejected, the fixed effects estimator is valid in this case.

In the following steps, model estimation was carried out with the fixed effects model in the group estimator method. Then, to ensure the model results, the assumptions of heteroscedasticity, autocorrelation, and inter-unit correlation, which are the basic assumptions of the panel regression models, were tested. If any of these assumptions occur, the t statistics and significance scores (*p*) cannot be trusted. In this direction, the Modified Wald test for heteroscedasticity assumption, LBT and Durbin Watson tests for autocorrelation hypothesis, and Pesaran CD tests for correlation between units were applied. The results of the tests showed that there are deviations in all three assumptions, so the current model cannot be used because it includes inter-unit correlation, autocorrelation, and heteroscedasticity. In heteroscedasticity, autocorrelation, and inter-unit correlation, the Driscoll and Kraay standard error correction estimator is one of the robust estimators (18). Therefore, Driscoll and Kraay's standard error correction estimator was used for the final estimation results of the study.

The final model estimation results are in Table 3 for static regression model.

**Table 3:** Panel data analysis fixed effect model estimate results.

**Variables**	**Coefficients**	***t*-statistic**	***p***
**The dependent variable: Risk of catastrophic health expenditures**			
Constant	40.0814 (3.170164)^*^	12.63	* < 0.001*
Pre	−0.3674226 (0.0412815)	−8.19	* < 0.001*
Income	−0.000948 (0.00044)	−6.97	* < 0.001*
Number of observations	578		
F statistic	61.65		
F prob.	0.000		
Method	Fixed-effects regression		
Within *R*-squared	0.18		

^*^Values in parentheses indicate the standard errors.

The *F* statistic found at 61.65 indicates that the model is statistically significant at the 1% significance level. The *R*-square value of the model shows that all explanatory independent variables have an explanatory power of ~18.8% in the risk of catastrophic health expenditures for surgical health services (Table 3).

The negative and statistically significant 1% level of the coefficient of the pre-variable in the model meets the expectations. This value can be interpreted as, keeping other variables constant, a one-unit increase in the rate of public health expenditures will reduce the risk of catastrophic health expenditure for surgical services by 0.36 units. In addition, the coefficient of the income variable, which is the explanatory variable in the model, was also negative and statistically significant at the 1% level. This finding also suggests a one-unit increase in the country's income level, holding other variables constant, is associated with a 0.0009 unit decrease in the risk of catastrophic health expenses for surgical services (Table 3).

Moreover, the model was tested using dynamic panel data method to estimate the effective factor on catastrophic health expenditure for surgical services between 2003 and 2019. The reason for using dynamic model as estimation method is the endogeneity or dynamism.

In Table 4, dynamic model Arellano-Bond GMM estimator results are presented using robust standard errors. Before the interpretation of the panel regression estimation results obtained in the analysis with the Generalized Moments Method (GMM), it is important to perform some consistency tests for the model. Three different tests were used for consistency. The Wald Chi2 test that tests the significance of the variables in the model as a whole, the Sargan test that tests the validity of the tools used in the model, and the Arellano-Bond (AB) autocorrelation tests that show whether the model has an autocorrelation problem.

**Table 4:** Arellano-bond robust standard errors GMM estimator results.

Arellano-bond dynamic model prediction Group variable: Countries Time: Years (2003–2019) Vehicle variable number: 35 Ω	Number of observations	578
	Number of groups	34
	Wald test	1,855.93
	*p*	* < 0.001*
**che**	**Coefficient**	**Std error**	***z***	***p***	**%95 confidence interval**	
che_t − 1_	0.472	0.032	13.43	* < 0.001*	0.479	0.654
pre	−0.307	0.111	−2.44	* < 0.001*	−0.417	−0.007
income	−0.081	0.028	−3.23	* < 0.001*	−0.129	−0.034
GMM: L(2/3).che						
Standard equation D.che D.pre D.income						
Sargan test statistic: 24.25435						
*P* for Sargan: 0.224						
AR (1): −2.7686 ve * < 0.001*						
AR (2): −0.51724 ve *>0.001*						

The model is statistically significant as a whole, according to the Wald test results. In addition, the relationship between instrument variables and error terms was tested with the Sargan test and it was concluded that the instrument variables were valid. The results of AR (1) shows that there is autocorrelation and AR (2) tests show that there is no autocorrelation problem as expected. When the obtained test results are evaluated collectively, it is concluded that the panel regression estimation results can be interpreted properly. The small sample correction suggested by Windmeijer (19) was made in the GMM estimates. ^*^, ^**^, ^***^ indicate 10, 5, and 1% significance levels, respectively (19). As excessive vehicle uses leads to deviating results, it is accepted as a rule of thumb that the number of vehicles should not exceed the number of units in GMM estimates. The Ω indicates that the number of vehicles is therefore limited. The descriptive statistical results of the models also show that there is no problem in the estimation of the models.

It is seen that all of the variables that are determinants of catastrophic health expenditure for surgical services are also significant at 1% confidence levels and the coefficients are consistent with expectations just like static regression model. As a result of both static and dynamic regression models, it is concluded that the share of public health expenditures in total health expenditures and per capita income calculated according to purchasing power parity have a negative statistically significant effect on the risk of catastrophic health expenditure for surgical services between 2003 and 2019 for 34 OECD countries.

## Discussion

Globally, although the share of out-of-pocket payments in health expenditures decreases, its share in income does not decrease because, in public health expenditures, states tend to establish an inclusive health system to prevent threats that may arise, especially for their citizens who are in poverty or at risk of poverty (20). It is an important approach to reduce the risk of catastrophic health expenditures by increasing the budget allocated to public health expenditures. The other approach, increasing the income level, can be interpreted as an issue with weak flexibility. Because it may take more time for countries to increase their income level than to increase the share of health expenditure in current income. However, as seen in the results of my study, it is seen that the effect of 1 unit of increase in health expenditure has a more significant effect (0.39) on the catastrophic health expenditure related to surgical procedures. Of course, the use for the difference in income level increase may not only be for health, which can also be considered a reason for the income level effect being low. In this case, it will be beneficial for every country that cares about health outcomes to increase the share of health expenditures in income (21). As Zhou and colleagues (22) mentioned in their research, it will be inevitable that the share of health expenditures in GDP will increase in the coming years due to the aging population. The fact that health expenditures are both low compared to countries with good health indicators and the share of health expenditures in the country's gross domestic product is low, increases the threat that catastrophic expenditures may pose. In addition, countries with high gross domestic product already have high health expenditures and health outcomes. For example, in OECD data for 2020, the countries with the highest health expenditures in terms of their share in GDP are the USA (18.8), Canada (12.9), Germany (12.8), France (12.2), and the United Kingdom (12.0) and the risks of catastrophic health expenditure in these countries are low (23, 24).

A study has shown that as the size of the pooled financial mechanism in healthcare financing increases, out-of-pocket expenditures decrease and the budget allocated to healthcare expenses effectively increases. From this perspective, out-of-pocket spending exhibits similar characteristics to catastrophic healthcare expenditures (25). On the other hand, Dash (26) examined the socioeconomic factors affecting health financing, covering the period of 2000–2013 in low and middle-income countries, and found that low tax revenues, low GDP per capita, and high debt service negatively impact health financing. Meanwhile, another study conducted between 1990 and 2014 using data from 15 major states in India, examined the dynamic relationship between macroeconomic factors such as health expenditures, economic growth, internal income, internal debt, fiscal balance, and central government transfers, showing that improvements in income, increases in tax base, and efficient use of central grants can create fiscal space in the economy and allow governments to allocate more funds to public health services (27). The results of this and my study are similar and coincide with the effect of the share of public health expenditures in total health expenditures and per capita income calculated based on purchasing power parity, reducing catastrophic health expenditure risk in the case of surgical procedures for the 34 OECD countries. On the other hand, an increase in positive macroeconomic factors such as taxes allocated to health financing, internal income, and economic growth can also increase health financing and reduce catastrophic health expenditures.

However, studies have shown that in terms of health outcomes, countries with less health expenditure will have a higher impact on the increase in health indicators for each unit of health expenditure that increases. In this case, another important question arises that needs to be discussed. Will the catastrophic health expenditure reduction results of 0.0003 in 1 unit of income level increase or 0.39 in 1 unit of health expenditure increase have a higher impact or how much impact will they have in developing countries? Boz et al. (28) found that Costa Rica and Turkey, which have a higher share of health expenditures in GDP than other countries, ranked highest in their studies assessing the risk of catastrophic health expenditures, some health indicators, and health systems in developing countries. It is seen that there is public inclusive health insurance in the health systems of both countries (29, 30). Doshmangir et al. (31) argued that setting an upper limit on catastrophic health expenses is necessary to avoid severe financial consequences related to the public coverage of treatment costs, as demonstrated in their systematic review and meta-analysis. However, further research using different health indicators and inputs could add to the existing literature on how to limit catastrophic health expenses and address other related questions.

## Data availability statement

The datasets presented in this study can be found in online repositories. The names of the repository/repositories and accession number(s) can be found in the article/supplementary material.

## Author contributions

The author confirms being the sole contributor of this work and has approved it for publication.
